# Majorana zero modes in the hopping-modulated one-dimensional *p*-wave superconducting model

**DOI:** 10.1038/srep17049

**Published:** 2015-11-20

**Authors:** Yi Gao, Tao Zhou, Huaixiang Huang, Ran Huang

**Affiliations:** 1 Department of Physics and Institute of Theoretical Physics, Nanjing Normal University, Nanjing, 210023, China; 2 College of Science, Nanjing University of Aeronautics and Astronautics, Nanjing, 210016, China; 3 Department of Physics, Shanghai University, Shanghai, 200444, China

## Abstract

We investigate the one-dimensional *p*-wave superconducting model with periodically modulated hopping and show that under time-reversal symmetry, the number of the Majorana zero modes (MZMs) strongly depends on the modulation period. If the modulation period is odd, there can be at most one MZM. However if the period is even, the number of the MZMs can be zero, one and two. In addition, the MZMs will disappear as the chemical potential varies. We derive the condition for the existence of the MZMs and show that the topological properties in this model are dramatically different from the one with periodically modulated potential.

Recently, searching for Majorana fermions (MFs) in condensed matter systems has attracted much attention^1^,^2^,^3^,^4^. MFs are their own antiparticles and in condensed matter systems, they can appear as quasiparticle excitations in topological superconductors. Because of their nonlocality and non-Abelian statistics, the zero-energy MFs, also called Majorana zero modes (MZMs) which refer to the zero-energy in-gap excitations, are proposed to be possible to realize fault tolerant topological quantum computation^5^,^6^,^7^. There are several suggestions of physical systems that may support the MZMs^8^,^9^,^10^,^11^,^12^,^13^, among which the one-dimensional *p*-wave superconducting (SC) model (also called the Kitaev model)^5^, due to its simplicity and elegance, is the most studied one. Possible realization of the Kitaev model includes quantum wires with a strong spin-orbit coupling (or topologically insulating wires subject to a Zeeman magnetic field) and in proximity to a superconductor^11^,^12^. In addition, it can also be realized in cold-atom systems^9^,^14^. Other proposals to realize the MZMs include ferromagnetic atomic chains placed in proximity to a conventional superconductor with strong spin-orbit coupling^15^ and atomic chains with a spatially modulated spin arrangement^16^,^17^,^18^,^19^,^20^.

Up to now, most of the theoretical works focus on ideal homogeneous^5^ or potential-modulated Kitaev chains^21^,^22^,^23^, or Kitaev chains with longer-range hopping and pairing^23^,^24^, or even quasi-one-dimensional Kitaev chains with a finite width^25^,^26^. Particularly in the periodically potential-modulated case^21^,^22^,^23^, it was found that under time-reversal symmetry, the number of the MZMs can be at most one and if the potential vanishes at certain sites, then the MZM will be very robust and stable for arbitrary strength of the modulation. However, a very important problem unaddressed is the stability and fate of the MZMs under hopping modulation. Naively people may speculate that they are similar under potential and hopping modulations. Whether this is true needs to be verified. Furthermore, if the two modulations result in different topological properties, we want to know what is new the hopping modulation can lead to. Therefore in this work, we investigate the hopping-modulated one-dimensional *p*-wave SC model which is an extension of the original Kitaev model. We found that, under time-reversal symmetry, the number of the MZMs strongly depends on the period of the modulation. If the period is odd, there can be at most one MZM. However if the period is even, in some parameter regimes the number of the MZMs can be two. Furthermore, the MZMs will disappear as the chemical potential varies no matter the period is odd or even. Therefore the topological properties of the hopping-modulated model are drastically different from those of the potential-modulated one.

## Method

We consider a one-dimensional Kitaev *p*-wave SC model where the hopping is periodically modulated, the Hamiltonian can be written as




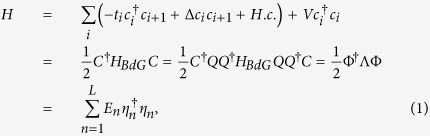




where 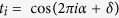 is the periodically modulated hopping integral. Δ ≠ 0 is the *p*-wave SC pairing gap and *V* is the chemical potential. Here *α* = *p*/*q* is a rational number with *p* and *q* being coprime integers. 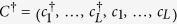 and 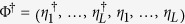, with *L* being the number of the lattice sites. In addition,




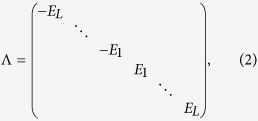




and *Q* is a unitary matrix that diagonalizes *H*_*BdG*_.

Defining Majorana operators 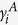 and 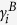 as




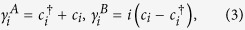




then the quasiparticle operator 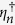 can be expressed as




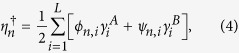




with *ϕ*_*n*,*i*_ and *ψ*_*n*,*i*_ being the amplitudes of the MFs 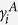 and 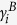 in the *n*th eigenstate, respectively. If there exist MZMs, then none of the *E*_*n*_ in Eq. (1) is zero under periodic boundary condition (PBC) while some of them become zero under open boundary condition (OBC) and the number of the MZMs is the number of the zero *E*_*n*_.

Since *t*_*i*_ is modulated with a period *q* (the unit cell is enlarged by *q* times), therefore under PBC, we have




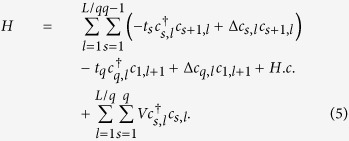




Using Fourier transform, 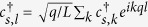, 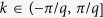. In momentum space, we get




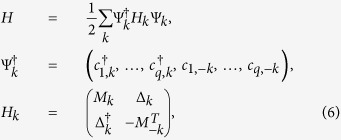




with the nonzero matrix elements of *M*_*k*_ and Δ_*k*_ being 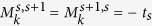 and 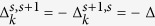, for 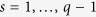. 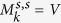 for 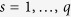, while 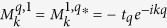 and 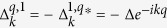.

Since we assume *t*_*i*_ and Δ in Eq. (1) is real (up to a global phase) throughout the paper, thus *H*_*k*_ respects the time-reversal, particle-hole and chiral symmetries and it can be unitarily transformed to an off-diagonal matrix as^26^,^27^,^28^




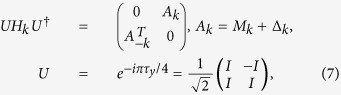




here *τ*_*y*_ is a Pauli matrix acting on the particle-hole space. Then the system belongs to the class BDI which is characterized by the 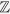 index while the number of the MZMs can be represented by *W* which is calculated through




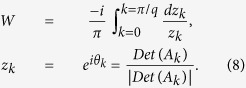




In fact, 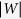 just counts how many times the determinant of *A*_*k*_ crosses the imaginary axis as *k* evolves from 0 to *π*/*q*.

## Results and Discussion

First we consider the *α* = 1/2 case, where *t*_1_ = −*t*_2_ = −cos*δ*. Under PBC, we have




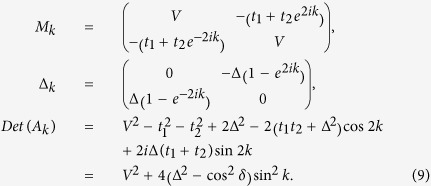




Since *Det*(*A*_*k*_) is real, *W* must be zero otherwise *Det*(*A*_*k*_) will be zero for some *k* (which means the bulk energy gap vanishes), therefore, there can be no MZMs.

Generally, the periodic modulation can take many forms. For arbitrary *t*_1_ and *t*_2_ (*t*_1_ ≠ −*t*_2_), we have




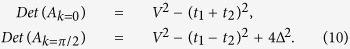




If *V* ≠ 0 and 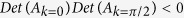, then *Det*(*A*_*k*_) will cross the imaginary axis once as *k* changes from 0 to *π*/2. In this case, 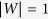 and one MZM exists. Interestingly, at *V* = 0, we have




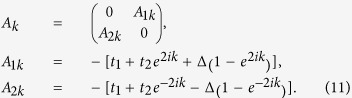




In this case, the system can be divided into two separated subsystems *A*_1*k*_ and *A*_2*k*_. As *k* evolves from 0 to *π*/2, we have




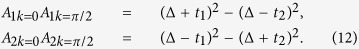




Therefore, if both 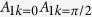 and 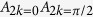 are less than zero, then two MZMs will show up in this case and the existence of these two MZMs has been numerically verified (for example, *t*_1_ = 0.5, *t*_2_ = −0.8 and Δ = 0.5).

**Figure 1:**
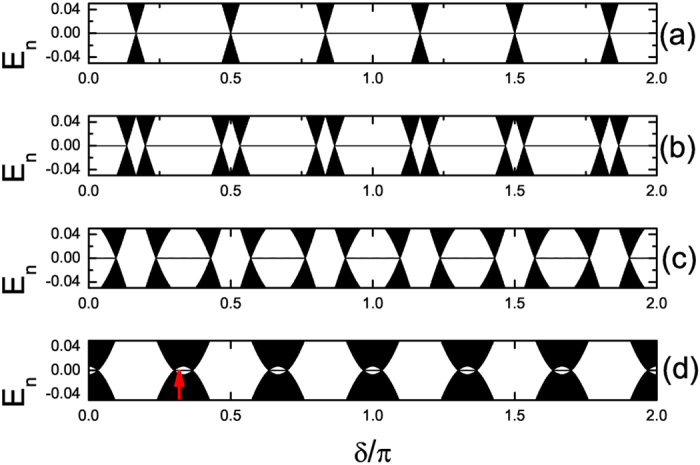
The energy spectra for *α* = 1/3 under OBC. Here Δ = 1 and *L* = 1632. (**a**–**d**) are for *V* = 0, 0.1, 0.2 and 0.3, respectively. Only 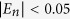 are plotted.

**Figure 2:**
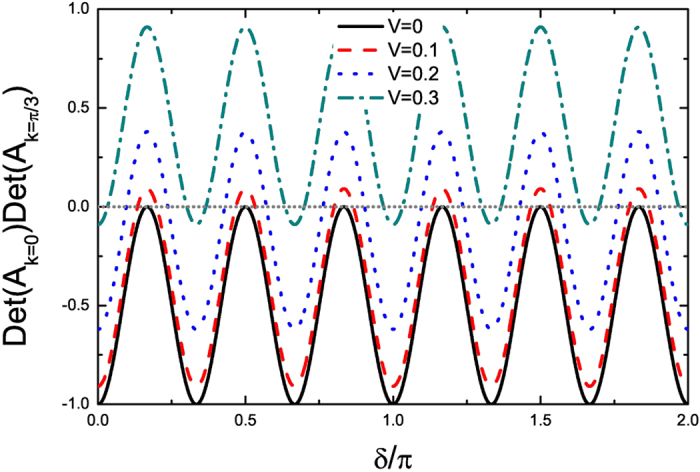
*Det*(*A*_*k*=0_)*Det*(*A*_*k*=*π*/3_) as a function of *δ* and *V*, for *α* = 1/3 under PBC. The black solid, red dashed, blue dotted and green dash dotted lines are for *V* = 0, 0.1, 0.2 and 0.3, respectively. The gray dotted line denotes *Det*(*A*_*k*=0_)*Det*(*A*_*k*=*π*/3_) = 0. Here Δ = 1 and *L* = 1632.

**Figure 3:**
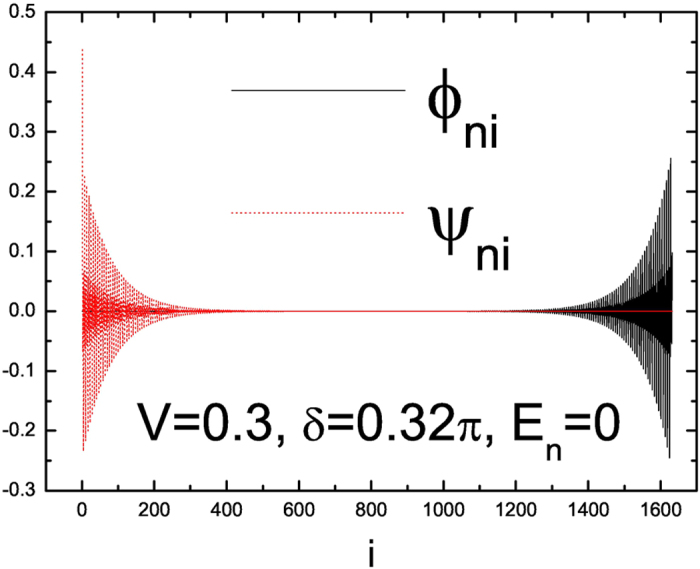
The distribution of the zero-mode MFs along the one-dimensional lattice for *α* = 1/3 under OBC. Here Δ = 1, *L* = 1632, *V* = 0.3 and *δ* = 0.32*π*, as denoted by the red arrow in Fig. 1(d).

For *α* = 1/3, we have




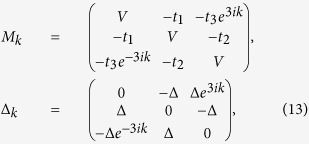




where *t*_1_ = cos(2*π*/3 + *δ*), *t*_2_ = cos(4*π*/3 + *δ*) and *t*_3_ = cos*δ*. In this case,




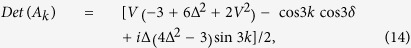




and 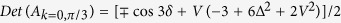 (− and + are for *k* = 0 and *π*/3, respectively). If 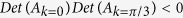, then 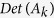 will cross the imaginary axis exactly once as *k* evolves from 0 to *π*/3, which means that 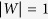 and one MZM exists in this case [*E*_1_ in Eq. (2) is zero under OBC]. On the contrary, 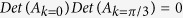 means that the bulk energy gap vanishes and 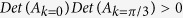 means that *Det*(*A*_*k*_) will not cross the imaginary axis as *k* evolves from 0 to *π*/3 in such a way that *W* = 0, in both cases no MZMs exist. Specifically, for *δ* = (2*m* + 1)*π*/6 with *m* being an integer, we have cos3*δ* = 0, therefore 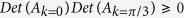 and no MZMs can exist, irrespective of the values of Δ and *V*. For example, we set Δ = 1 and *L* = 1632. In Figs 1 and 2 we plot the energy spectra under OBC and 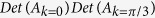 under PBC, respectively. As we can see, at *V* = 0, MZM exists for any *δ*, except for *δ* = *π*/6, *π*/2, 5*π*/6, 7*π*/6, 3*π*/2, 11*π*/6 where the bulk energy gap closes. As *V* increases, for some *δ*, MZM vanishes and as *δ* evolves from 0 to 2*π*, topologically trivial (without MZM) and nontrivial (with one MZM) phases appear in turn. A typical distribution of the zero-mode MFs is shown in Fig. 3 and we can see that the two MFs 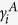 and 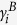 are well separated in real space and are located at the left and right ends, respectively while the actual decay length of these two MFs increases/decreases as the bulk energy gap decreases/increases. Finally when *V*  > 0.32 where the condition 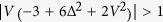 is satisfied, MZM disappears for any *δ* and there is only topologically trivial phase and indeed for *δ* = (2*m* + 1)*π*/6 with *m* being an integer, MZMs do not exist for any *V*.

For *α* = 1/4, we have *t*_1_ = −*t*_3_ = −sin*δ* and *t*_2_ = −*t*_4_ = −cos*δ*. In this case,




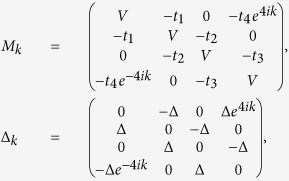







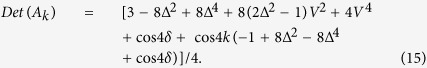




**Figure 4:**
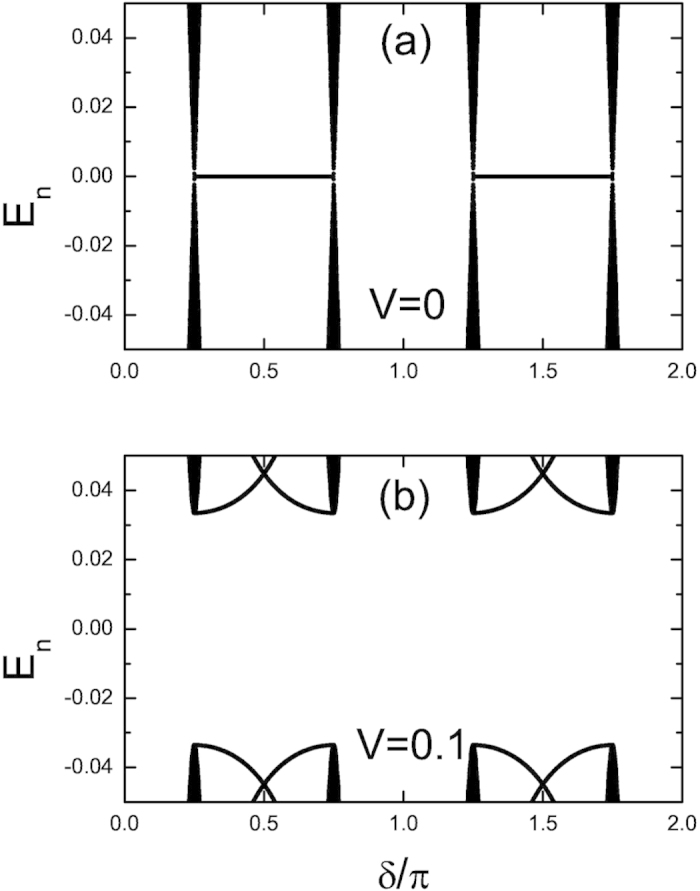
The energy spectra for *α* = 1/4 under OBC. Here Δ = 1 and *L* = 1632. (a) *V* = 0. (b) *V* = 0.1. Only 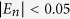 are plotted.

**Figure 5:**
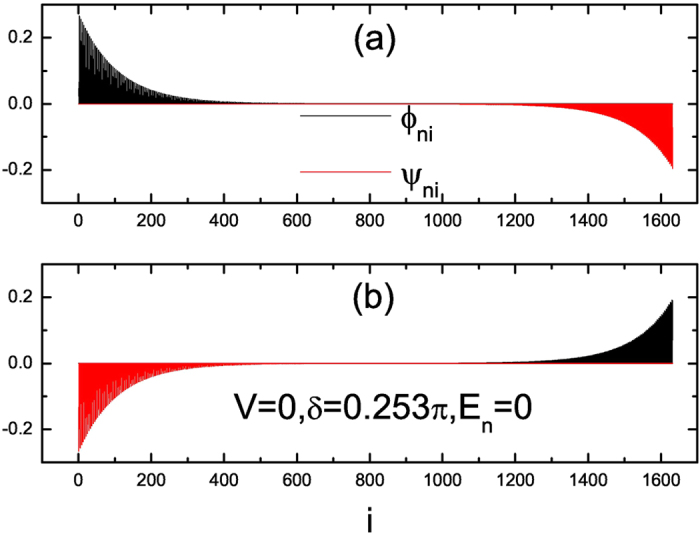
The distribution of the zero-mode MFs along the one-dimensional lattice for *α* = 1/4 under OBC. Here Δ = 1, *L* = 1632, *V* = 0 and *δ* = 0.253*π*. (a) The eigenstate corresponding to *E*_1_ = 0. (b) The eigenstate corresponding to *E*_2_ = 0.

At first glance, since *Det*(*A*_*k*_) is real, thus, similar to the *α* = 1/2 case, there should be no MZMs. However, this is not true at *V* = 0. In the following we set Δ = 1 and *L* = 1632 as an example. As we can see from Fig. 4, indeed at *V* = 0.1, there are no MZMs. However at *V* = 0, MZMs exist for *π*/4 < *δ* < 3*π*/4 and 5*π*/4 < *δ* < 7*π*/4. These MZMs are doubly degenerate [both *E*_1_ and *E*_2_ in Eq. (2) are zero under OBC] and the distribution of the zero-mode MFs is shown in Fig. 5. The existence of these two MZMs can be explained as follows. At *V* = 0, we found that the eigenvalues of *H*_*k*_ in Eq. (6) are doubly degenerate, therefore *H*_*k*_ can be divided into two independent subsystems by a unitary transformation as




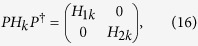




here both *H*_1*k*_ and *H*_2*k*_ are 4 × 4 matrices while their eigenvalues are exactly the same. The unitary matrix *P* can be written as




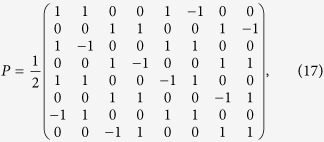




and




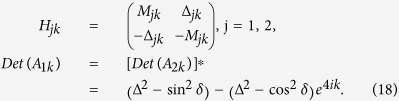




At *k* = 0, 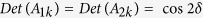 while at *k* = *π*/4, 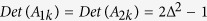. If 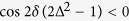, then both *Det*(*A*_1*k*_) and *Det*(*A*_2*k*_) will cross the imaginary axis exactly once as *k* evolves from 0 to *π*/4, indicating that there exists one MZM in each subsystem and the number of the MZMs for the whole system is two. Therefore for *α* = 1/4, at *V* = 0, the number of the MZMs are either two or zero while at *V* ≠ 0, there are no MZMs. For general *t*_*i*_ 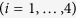, at *V* ≠ 0, *Det*(*A*_*k*_) may not be real and there may exist one MZM. However at *V* = 0, the system can still be divided into two subsystems. In this case, if the conditions 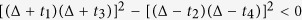 and 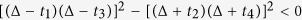 are satisfied simultaneously, there will be two MZMs.

Furthermore we found that, for general periodic modulation, if the period *q* is odd, then the number of the MZMs is either zero or one. On the other hand, if *q* is even, then at *V* ≠ 0, the number of the MZMs is still zero or one. However at *V* = 0, the system can always be divided into two independent subsystems and if the conditions




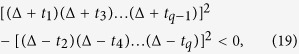




and




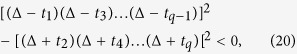




are simultaneously satisfied, there will be two MZMs.

In summary, we have studied the number of the MZMs and their stability in the hopping-modulated one-dimensional *p*-wave SC model. We found that the former strongly depends on the period of the modulation. If the period *q* is odd, there can be at most one MZM in the system while for an even *q*, the number of the MZMs can be zero, one and two. The existence of two MZMs can occur only at *V* = 0, since in this case, *A*_*k*_ in Eq. (7) can always be divided into two independent sub-matrices by a unitary transformation as




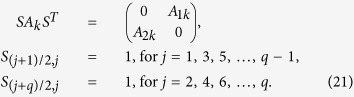




At certain conditions, there exists one MZM in each subsystem and the number of the MZMs for the whole system is two. If *A*_1*k*_ can be further separated into two subblocks by another unitary transformation *R* (which is *k*-independent and real, up to a global *k*-independent phase) as




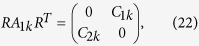




then after a tedious calculation we can prove that *Det*(*C*_1*k*_) and *Det*(*C*_2*k*_) cannot be complex simultaneously and they can cross the imaginary axis at most once as *k* varies from 0 to *π*/*q*. Therefore, even if *A*_1*k*_ can be further separated into two subblocks *C*_1*k*_ and *C*_2*k*_, only one of them may host one MZM, making the maximal number of the MZMs in *A*_1*k*_ be one. The same argument can be applied to *A*_2*k*_ as well. Thus for an even modulation period, there can be at most two MZMs. For the specific modulation form we considered [*t*_*i*_ = cos(2*πiα* + *δ*) with *α* = *p*/*q*], only at *q* = 4*n* 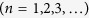 can Eqs (19) and (20) be simultaneously satisfied, therefore only in this case can there exist two MZMs. Furthermore, the MZMs will vanish as the chemical potential *V* varies. In the periodically potential-modulated model considered in Refs. 21, 22, 23, when the time-reversal symmetry is present, there can be at most one MZM and if the potential vanishes at certain sites, then the MZM will be very robust and stable for arbitrary strength of the modulation. Clearly this is not the case in the periodically hopping-modulated model, therefore the topological properties differ drastically between these two models.

At last we would like to emphasize the motivation as well as the physical implications of our study. As we know, exploring various topological properties in different models is of both fundamental and practical importance. From the fundamental point of view, it may help people to understand the mechanism and condition for the existence of the MZMs. As stated in the introduction section, intuitively people may speculate that the topological properties are similar between the hopping-modulated and potential-modulated Kitaev models. However in fact this is not the case as has been demonstrated in our study where both the number and stability of the MZMs differ drastically between these two models and these different behaviors have never been reported before. Furthermore we have demonstrated that, for multiband systems, special caution has to be taken when calculating the number of the MZMs from the 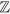 index. That is, when the system can be separated into two subsystems, the number of the MZMs may be mistakenly thought to be zero while there are actually two MZMs. On the other hand, from the practical point of view, our work, together with those previous studies concentrating on the potential modulation, may help to guide researchers to fabricate various topological phases with different numbers of the MZMs and to further manipulate them in order to realize topological quantum computation. We expect that our model is most likely to be realized in cold-atom systems and in optical superlattices where the hopping can be adjusted. In solid state devices, the direct modulation of hopping may be difficult. However we notice, the *p*-wave Kitaev model corresponds to the transverse spin model as^29^




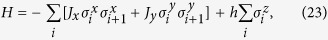




with 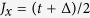, 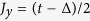 and *h* = −*V*/2. Therefore the chemical potential *V* = 0 can be achieved by setting *h* = 0 (zero Zeeman field). Furthermore, since *t* = *J*_*x*_ + *J*_*y*_ and Δ = *J*_*x*_ − *J*_*y*_, the modulation of hopping may be possible if *J*_*x*_ + *J*_*y*_ varies in space while *J*_*x*_ − *J*_*y*_ is constant. This may be realized in atomic chains with a spatially modulated spin arrangement (see refs. 16, 17, 18, 19, 20). Therefore the ideas in our work are both fundamentally sound and practically applicable.

## Additional Information

**How to cite this article**: Gao, Y. *et al.* Majorana zero modes in the hopping-modulated one-dimensional *p*-wave superconducting model. *Sci. Rep.* **5**, 17049; doi: 10.1038/srep17049 (2015).
